# Special Issue: Examining the Impacts of Social Determinants on HIV Health and Prevention

**DOI:** 10.3390/ijerph21010030

**Published:** 2023-12-25

**Authors:** K. B. Boomer

**Affiliations:** Department of Mathematics, Bucknell University, Lewisburg, PA 17837, USA; kb.boomer@bucknell.edu; Tel.: +1-570-577-1915

Assessing the impact of social determinants of health (SDoH) has become an important part of the holistic view of an individual’s health status. The World Health Organization (WHO) has defined social determinants of health as “the conditions in which people are born, grow, live, work and age. These circumstances are shaped by the distribution of money, power and resources at global, national and local levels” [1]. The WHO describes the HIV pandemic as a “pandemic driven by inequalities” [2]. In 2016, the General Assembly called for 90% of people living with HIV (PLHIV) to know their status, 90% to be accessing antiretroviral therapy, and 90% to be virally suppressed [3]. The barriers to accessing care related to SDoH include finances, unemployment, physical activity, and health literacy [4,5].

This Special Issue brings together many leading scholars, including members of the National Working Positive Coalition’s Research Working Group, to examine key social determinants of HIV health and prevention. Addressing these SDoH is essential for improving overall wellbeing and health outcomes for PLHIV. A prominent theme throughout many of the articles is the way in which economic factors impact HIV health and prevention. Finances can be a substantial barrier to remaining in care and being virally suppressed [6]. In a 2017 study, Wohl et al. examined patients receiving AIDS Drug Assistance Programs (ADAPs), which are intended to provide medications at no cost or low cost. The study found that 21% of ADAP recipients failed to maintain ARV medication due to lapses in coverage or cost.

The first article in the Special Issue, “Examining Cash Expenditures and Associated HIV-Related Behaviors using Financial Diaries in Women Employed by Sex Work in Rural Uganda: Findings from the Kyaterekera Study”, explores the financial lives of women employed in sex work through financial diaries. These diaries show that the primary expenditures were for food, sex work expenditures, and housing. Only five percent of expenditures were on healthcare costs; less than half the participants were on either ART or PrEP medications.

The article “Examining the Implementation of Conditional Financial Incentives Using the Reach, Effectiveness, Adoption, Implementation, and Maintenance (RE-AIM) Framework to Improve HIV Outcomes among Persons Living with HIV (PLWH) in Louisiana” used an economic strengthening approach with financial incentives, with the goal of increasing retention in care or helping individuals begin engagement in care, as well as maintaining medical adherence and a reduction in viral load. This aligns with the goals of the HIV Continuum of Care outcomes [7]. As reported in the article, the participants were given up to USD 140 in incentives for having their viral load checked at up to three time points (baseline, 6-month follow-up, 12-month follow-up) and remaining virally suppressed. Chrestman et al. reported that engagement in care was high (93%) at the third time point, although viral suppression decreased from 59% at baseline to 34% at the final time point. Structural factors, such as employment, housing, and having health insurance, were not associated with engagement or retention in care. Chrestman et al. suggest that one reason for the insignificant findings may be that the structural factors are too complex to be offset by the low amount offered as a financial incentive.

In “Socioeconomic Status and the Sense of Coherence among Japanese People Living with HIV”, the authors explore the idea of a sense of coherence (SOC) with common stressors facing PLHIV, including financial stressors. SOC is a component of salutogenesis, and those with a strong SOC are believed to be more able to cope with these stressors and thus achieve better health [8]. Noting that education level, occupation, and income are strongly associated in Japan, Togari et al. report that a lower socioeconomic status is associated with a lower SOC. “Unemployed job seekers” and those who reported their occupation as “homemaker/recuperating from an illness/student” were more likely to report a low sense of coherence than the reference level (freelancer). These data show that 96.3% of the participants aged 25 and older classified themselves as “homemaker/recuperating from an illness/student”, which Togari et al. suggest means a large proportion of PLHIV in Japan do not work as they are focusing on their medical needs. A lower income was associated also associated with a lower sense of coherence, raising concerns about the health prospects of lower-income PLHIV.

In “The Role of the Social Determinants of Health onEngagement in Physical Activity or Exercise among Adults Living with HIV: A Scoping Review”, Safa et al. reviewed 41 articles and found that women were less likely to engage in physical activity than men. There was no association found between unhealthy behaviors such as smoking and physical activity. Importantly, this review points out some gaps in the literature relating to physical activity and PLHIV. Notably, none of the articles reviewed used a SDoH framework, and there is little consistency in how physical activity was determined and measured. Additionally, most of the studies were cross-sectional, highlighting the need for more longitudinal studies to establish causal relationships.

Research suggests that employment is positively linked with several outcomes in the HIV Continuum of Care, namely HIV testing, linkage to HIV care, retention in HIV care, and HIV medication adherence [5]. Additionally, studies show that employed PLHIV have a higher health-related quality of life than those who are unemployed [9]. In “Measuring Phases of Employment Decision-Making and the Need for Vocational Services as a Social Determinant of the Health of Employed People Living with HIV”, Boomer et al. explore the impact of employment through a newly developed Considering Work scale for employed PLHIV. This scale helps to classify individuals as contemplating or planning a change in employment, preparing for a change, actively making a change, or resolving not to change. The study also found high levels of insecure employment among PLHIV, with nearly half the sample unsure of whether they could maintain their current employment.

In “Psychosocial and Health-Related Behavioral Outcomes of a Work Readiness HIV Peer Worker Training Program”, McKinney-Prupis et al. report the positive outcomes associated with participation in an HIV peer worker training program. Peer HIV education has been shown to be effective due to the shared lived experience [10]. In this Special Issue article, the peer education curriculum included HIV-related content, safer sex practices, substance abuse, and harm reduction. McKinney-Prupis reports that the program participants demonstrated positive health-related behavioral outcomes such as medication adherence, increased self-advocacy, and greater confidence in discussing safer sex practices. In addition, participants reported a reduction in depression symptoms and internalized HIV stigma and increased self-esteem. This study underscores the important role that vocational training for peer workers can play in overall HIV health and prevention for participants. 

Related to HIV education, the article “Responding to the HIV Health Literacy Needs of Clients in Substance Use Treatment: The Role of Universal PrEP Education in HIV Health and Prevention” focuses on the SDoH of health literacy in people with substance-use disorders (PWSUDs), as this group is susceptible to HIV infection. Pre-exposure prophylaxis for HIV (PrEP) has been shown to be effective at preventing HIV transmission, but many counselors lack the knowledge to share the benefits of PrEP with their PWSUD clients. The authors cite six contributors to a lack of awareness and use of PrEP: a lack of HIV health literacy at both the individual and organizational levels, financial barriers, geographical barriers, housing instability, and stigma. The article presents recommendations for providing PrEP education and use within a trauma-informed framework. Given the negative impact of chronic health conditions on economic status and quality of life, health education that can prevent HIV is an important social determinant of health for PWSUDs that needs more attention. 

This Special Issue would not be possible without the commitment of all the authors who contributed their time and expertise. It was a pleasure to serve as a final reviewer for each of the articles in collaboration with the co-editor for this issue, Dr. Liza Conyers. We are grateful for this opportunity and appreciate the efforts of all who contributed to this Special Issue despite the impact of COVID-19. The authors and their affiliations represent key collaborations among researchers, community partners, and policy makers. 
